# Defining treatment-resistant brain cancer: Genetic screening to identify oncogene-driven immunomodulation and therapy resistance

**DOI:** 10.1038/s41417-025-00980-y

**Published:** 2025-10-26

**Authors:** Zachary David Myers, Sandro Matosevic

**Affiliations:** 1 Department of Industrial and Molecular Pharmaceutics, Purdue University, West Lafayette, IN USA; 2 Institute for Cancer Research, Purdue University, West Lafayette, IN USA

**Keywords:** Cancer genomics, CNS cancer

## Abstract

Glioblastoma multiforme (GBM) is an extremely aggressive brain tumor characterized by rapid progression, poor prognosis, and limited potential for remission. A contributing factor to the aggressiveness of GBM is the high genetic and phenotypic variability of the tumor caused by the accumulation of beneficial mutations over time. Screening methodologies utilizing genetic tools such as clustered regulatory interspaced short palindromic repeats (CRISPR) and ribonucleic acid (RNA) interference (RNAi) have proven effective in identifying oncogenic driver genes in GBM. Here, we analyze and summarize these studies. Analysis of hits emerging from genetic screens in GBM has revealed key factors with the capacity for regulating deoxyribonucleic acid (DNA) repair, cell cycle, or metabolism of the cancer. The genetic programs which endow GBM a high degree of aggressiveness also contribute to outcompeting immune cells associated with tumor cell clearance. Genes identified in genetic screens influence the receptor landscape on the surface of both GBM and immune cells, as well as the soluble factors within the tumor microenvironment (TME). These soluble factors and surface receptors regulate function of immune cells, in particular natural killer (NK) cells. This systematic review links genetic drivers of GBM identified through screening approaches and their documented roles. As will be discussed, these genes were shown in literature to encode molecular programs that confer a competitive advantage to GBM in contexts such as chemotherapy and radiotherapy.

## Introduction

Despite the variability in oncogenic factors across different cancers, there are well-established fundamental hallmarks that characterize cancerous tissues. Altered metabolism, replicative immortality, aberrant growth, and immunological evasion have been described as characteristics of tumors regardless of type. More recently, the importance of epigenetic reprograming and the TME have been implicated in cancer identity and progression [1–3]. While numerous genes may fulfill these prototypical cancer attributes, each cancer exhibits unique genetic characteristics, even within the same pathological subtype [4]. One such genotypically and phenotypically heterogeneous tumor is GBM, an aggressive brain tumor. Despite aggressive standard of care prompting surgical resection, radiotherapy and chemotherapy, the five year survival has stagnated at around 7.2% [5–7]. GBM is characterized by severe immunosuppression. Additionally, there is increasing evidence suggesting oncogenes potentiate immune escape and evasion [8]. Effector cells, such as NK cells, which serve as an innate defense mechanism against cancer, demonstrate suppressed function in controlling GBM. This suppression is; due in part, from specific driver genes found in cancer cells [9]. Despite this, efforts to target cancer with therapeutic doses of NK cells have progressed to phase two clinical trials; albeit with the greatest responses registering for cancers of the blood rather than solid tumors [10]. Similarly, efforts to genetically engineer NK cells are being pursued to better combat immunosuppression induced by cancer cells and the TME [11–13]. To determine molecular drivers of cancer, genetic screens have aided in elucidating genes regulating various processes such as cancer initiation, proliferation, and metastasis. Among these, RNAi and CRISPR genetic screens have been shown to be particularly powerful and effective [14, 15].

In this systematic review, we summarize genomic screens in GBM to highlight key genes which have emerged from such screening efforts. Reviews currently exist which have established primary findings of genetic screens. In this work we categorize actionable hits from these genomic screens as being related to DNA repair and immortality, cell cycle, or metabolic processes. We further discuss published studies evidencing each gene’s contribution to GBM. While all genes discussed were derived from genetic screens in GBM, their molecular relevance to cancer may have been determined in a different tumor type. In addition, the impact of each gene on NK cell function is explored, and attributed to either differences in NK cell surface receptor expression or alterations in the TME.

## Identifying and characterizing genetic drivers of GBM from genetic screening studies

**Fig. 1:**
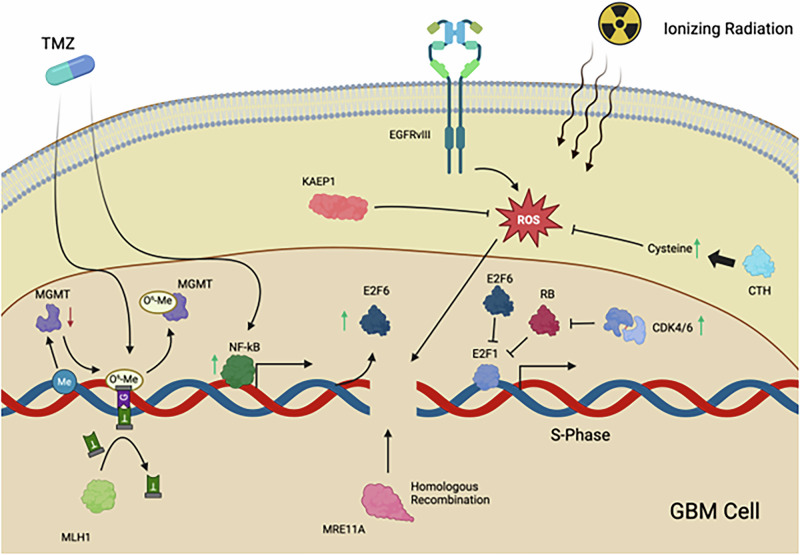
Molecular functions of metabolic, cell-cycle regulation, and DNA-repair driver genes identified through genetic screens in GBM. TMZ treatment induces O^6^-Me guanine adducts which facilitate guanine and thymine pairing. While O^6^-Me guanine adducts are typically removed from guanine by the protein MGMT, in EGFRvIII-mutant GBM, DNA methylation frequently downregulates MGMT transcription. In the absence of MGMT, futile cycling of the MMR proteins MLH1 and MSH2 are not able to resolve TMZ-induced DNA damage [99, 100]. In many GBM tumors, elevated CDK4/6 activity drives persistent S-Phase, which is suppressed by E2F6, whose expression is induced by elevated NF-kB levels during TMZ treatment [28, 30, 74]. Both EGFRvIII and ionizing radiation drive elevated ROS levels in GBM cells which are suppressed by KAEP1 and indirectly through CTH-mediated cysteine production [79, 82, 83, 89, 178, 211]. Downstream, ROS facilitate DNA damage which, when in the S-phase, may be repaired through the HR protein MRE11A [57, 212].

To characterize the hits from various genetic screening studies in GBM, we categorized selected genes into one of three categories. These categories include DNA repair and damage response, control of cell cycle and immortality, or mediators of metabolism (Fig. 1). In several cases, the genes were characterized as such in the original paper – in other cases, we made such determination (Table 1). It is worth noting that genes may belong under multiple categories. However, to avoid redundancy within this review, each gene was assigned to only one of the three categories mentioned.

**Table 1:** Genes identified as regulators of GBM processes from genetic screens carried out in the literature.

Gene	Categorical classification	Screen type used to identify	CRISPR approach	Reference
***BRCA2***	DNA Repair	OCT Perturb-Seq	CRISPRi	[40]
***ERCC4***	DNA Repair	OCT Perturb-Seq	CRISPRi	[40]
***LIG4***	DNA Repair	OCT Perturb-Seq	CRISPRi	[40]
***Mre11a***	DNA Repair	OCT Perturb-Seq	CRISPRi	[40]
***PRKDC***	DNA Repair	OCT Perturb-Seq	CRISPRi	[40]
***BORA***	Cell Cycle and Immortality	OCT Perturb-Seq	CRISPRi	[40]
***HSD17B10***	Metabolism	OCT Perturb-Seq	CRISPRi	[40]
***CYP19A1***	Metabolism	OCT Perturb-Seq	CRISPRi	[40]
***YY1***	Cell Cycle and Immortality	Non-targeted CRIPSR Screen	CRISPR KO	[66]
***PKMYT1***	Cell Cycle and Immortality	Non-targeted CRIPSR Screen	CRISPR KO	[67]
***LRP8***	Metabolism	Biased Genome-Wide Screens	CRISPRa	[72]
***TERT***	Cell Cycle and Immortality	Biased Genome-Wide Screens	CRISPR KO	[71]
***E2F6***	Cell Cycle and Immortality	Biased Genome-Wide Screens	CRISPR KO	[74]
***KEAP1***	Metabolism	Targeted CRISPR Screen	CRISPR KO	[78]
***NDUFS8***	Metabolism	Targeted CRISPR Screen	CRISPR KO	[79]
***SMPD1***	Metabolism	Targeted CRISPR Screen	CRISPR KO	[79]
***COMTD1***	Metabolism	Targeted CRISPR Screen	CRISPR KO	[79]
***CSE***	Metabolism	Targeted CRISPR Screen	CRISPR KO	[79]
***SMS***	Metabolism	Targeted CRISPR Screen	CRISPR KO	[79]
***HDAC2***	Cell Cycle and Immortality	Targeted CRISPR Screen	CRISPR KO	[78]
***MAP4K4***	Cell Cycle and Immortality	Targeted CRISPR Screen	CRISPR KO	[80]
***MET***	DNA Repair	Targeted CRISPR Screen	CRISPR KO	[78]
***ZC3H7A***	DNA Repair	Targeted CRISPR Screen	CRISPR KO	[64]
***MCM8***	DNA Repair	Targeted CRISPR Screen	CRISPR KO	[64]
***MCM9***	DNA Repair	Targeted CRISPR Screen	CRISPR KO	[64]
***MLH1***	DNA Repair	Targeted CRISPR Screen	CRISPR KO	[64]
***MSH2***	DNA Repair	Targeted CRISPR Screen	CRISPR KO	[64]
***USP21***	Metabolism	RNAi Screen	N/A	[101]
***CHMP2A***	Cell Cycle and Immortality	TCT Screen in GBM	CRISPR KO	[104]

### Mediation of metabolism

Altered metabolism in GBM, in comparison to healthy tissue, is well documented [16]. GBM is believed to rely on classical Warburg metabolism, wherein adenosine triphosphate (ATP) is predominantly generated through the conversion of glucose to lactate, even in the presence of oxygen. Additionally, in GBM, reversal of the Warburg effect has been shown to impair tumor growth and proliferation [17, 18]. Increased expression of the glucose transporter 1 (Glut1) has been observed in the core of GBM tissues compared to tumor periphery, with Glut1 levels correlating with lower patient survival [19, 20]. The expression of α-ketoglutarate (α-KG), a key metabolite, has been linked to N6-methyladenosine (m6A) modifications, which play an important role in cellular metabolism and gene regulation. These m6A modifications are partially mediated by methyltransferase-like 3 (METTL3). This regulation has been attributed to driving glioblastoma stem cell (GSC) stemness [21]. This evidence has established an intersection between metabolic pathways and GSC stemness in regulating GBM proliferation. In contrast, the presence of iron chelators has been found to decrease GBM proliferation [22]. However, competency in iron regulation has been shown lead to higher lipid peroxidation levels, which can ultimately trigger ferroptosis [23]. To combat ferroptosis, GBM cells rely on cystine uptake through solute carrier family 7 member 11 (SLC7A11) to drive glutathione (GSH) peroxidase 4 (GPX4)-dependent reduction of peroxidized lipids [24]. However, altered lipid metabolism has been shown to impact GBM morphology and contribute to the conferral of temozolomide (TMZ) resistance to GBM cells [25]. As will be discussed, genes identified through genomic screens involving GBM which were identified as being related to metabolic processes, were largely associated with regulation of reactive oxygen species (ROS) levels, glycolytic metabolism and lactate accumulation.

### Control of cell cycle and immortality

GBM may, in part, be characterized by mechanisms which alter cell cycle progression, proliferation, survival, and replicative immunity. Several molecular drivers contributing to cell cycle alterations have been described. Among these is the receptor tyrosine kinase (RTK)/Rat sarcoma virus (RAS)/ phosphoinositide 3-kinase (PI3K) pathway, which drives proliferation and survival, in part, in a mammalian target of rapamycin (mTOR)-dependent manner [26]. Retinoblastoma (Rb) protein, on the other hand, has been associated with mediating timely control of cell cycle progression from the G1 to the S phase [27]. Moreover, Rb has been established as a repressor which canonically complexes with transcription factor E2F (E2F) family members and suppresses progression into the S phase. Mutations and alteration in Rb along with those in the RTK/RAS/PI3K signaling pathways were found in 78% and 88% of GBM cases, respectively. Mutations in the Rb pathway include deletions in *CDKN2A*, which encodes p16, and amplifications of *CDK4*. These alterations facilitate Rb phosphorylation and S-phase progression [28–30]. In addition, in GBM, significant increases in *CDK1* levels have been associated with higher *MTOR* and *MYC* levels. Cyclin-dependent kinase (CDK) 1 plays a role in G2/M checkpoint control, whereas MYC regulates cancer cell growth and survival [31, 32]. Over 50% of all glioma cases have been reported to have *TERT* promoter mutations. These mutations contribute to replicative immortality, supporting aberrant cell growth and cell cycle regulation mechanisms in GBM [33]. This collective evidence demonstrates that regulation of cell cycle progression and mechanisms fostering replicative immunity contribute to GBM pathology. Consequently, many genes identified through genetic screening are involved with these pathways, which are commonly mutated in GBM.

### DNA repair and damage response

The progression of GBM relies, in part, on accumulation of genetic mutations [34]. Among these, *PIK3R1* mutations were found in nearly 10% of GBM incidences, specifically impacting the PI3K/protein kinase b (AKT)/mTOR pathway which mediates cancer cell growth, survival and DNA repair in response to DNA damage. Genetic mutations associated with defects in DNA repair machinery have been shown to be related to homologous recombination (HR) and mis-match repair (MMR) proteins [35]. However, the mutations that confer competitive advantages to cancer cells may act as double-edged swords, by making the cancer more susceptible to chemotherapeutics and radiation. Studies have suggested that in response to cisplatin, cancer cells deficient in *BRCA*, a gene involved in HR, show significantly more chromosomal abnormalities than wild type (WT) cells [36]. Cisplatin is a small-molecule cancer therapeutic which induces DNA damage. Together, this evidence suggests that while mutations or inadequacy of DNA repair mechanisms may play a role in promoting cancer neogenesis and progression, these deficiencies may increase sensitivity of cancer to therapy. It is important to note that the genes discussed and categorized as DNA repair and damage response genes do not include those which promote the transition of healthy to cancerous cells. Rather, the following genes refer to those which were found to sensitize, or desensitize, GBM to chemo- or radiation therapy.

## Genetic screen-based identification of gene regulators of GBM

### One cell type (OCT) Perturb-Seq

OCT targeted screens are defined as genetic screens performed in a single cell line with a focused gene library, as opposed to being genome wide [37, 38]. In perturb-Seq, an initial perturbation, typically in the form of a CRIPSR-based modification, is performed, and this is followed by sequencing via single cell RNA seq (scRNA-seq) [39]. So far, one Perturb-Seq study has been reported in human GBM. In this study, CRISPR interference (CRISPRi) screening was performed to narrow down genetic candidates which demonstrated resistance or sensitivity to radiotherapy. The selected candidate perturbations were followed by scRNA-seq. This resulted in eight genes for which individual knockouts led to differential expression of over one-hundred other genes following targeted library analysis, in response to radiotherapy. These genes included *BRCA2, ERCC4, LIG4, Mre11a, PRKDC, BORA, HSD17B10*, and *CYP19A1* [40].

### Mediation of metabolism

#### CYP19A1

In a Perturb-Seq screen of human GBM cells treated with radiotherapy, *CYP19A1*, which encodes aromatase (CYP19A1), was found to be among the most differentially expressed genes over normal conditions. [41] Recent studies have suggested that CYP19A1 facilitates upregulation of 17β-estradiol levels, thereby potentiating control of oxidative stress associated with TMZ treatment. It was also observed that nuclear factor erythroid 2-related factor (Nrf2) was necessary for TMZ resistance [42]. Nrf2 has been associated with significant increases in superoxide dismutase (SOD)1 and SOD2, which are mediators of oxidative stress [43, 44]. These findings suggest mechanisms for maintaining oxidative stress in GBM.

#### HSD17B10

*HSD17B10* emerged as another Perturb-Seq target from a screen of human GBM cells exposed to radiotherapy. Though it had previously been established that, in prostate cancer bone metastases, 17-β-hydroxysteroid dehydrogenase 10 (HSD17B10) levels were elevated [45],. limited studies have described the role of *HSD17B10* in GBM. Mechanistically, acetylation of HSD17B10 has been shown to decrease its enzymatic activity as a dehydrogenase critical for maintenance of mitochondrial function. Findings have also suggested that stresses such as hydrogen peroxide (H_2_O_2_) lead to increased Sirtuin 3 (SIRT-3), and subsequently decreased HSD17B10 acetylation. Moreover, it was determined that HSD17B10 knockdown mediates decreased cell survival and resistance to stress [46]. Therefore, *HSD17B10*, which encodes HSD17B10, has been shown to play a role in mediating oxidative stress, which may have metabolic implications.

### Control of cell cycle and immortality

#### BORA

*BORA* emerged as another target from Perturb-Seq analyses of radiotherapy-treated GBM. While *BORA* is not well characterized as a prognostic factor in GBM, protein aurora borealis (BORA) has been identified as a poor prognostic factor in adenocarcinoma [47]. *BORA* deficiency has been associated with aberrant mitotic processes. Furthermore, it has been established that BORA, a nuclear protein, may be shuttled to the cytoplasm by Cdk 2, thereby facilitating Aurora A activation [48]. Together, this situates *BORA* as a potential regulator of cell cycle progression in GBM.

### DNA repair and damage response

#### BRCA2

*BRCA2* has not typically been associated with low mutational burden in GBM, with expression of *BRCA1* and *BRCA2* mutant GBM found in 1.4% of patient cases [49]. However, in other cancers, such as breast cancer, *BRCA2* is a well-established oncogenic driver. It has been reported that *BRCA2* is responsible for controlling HR mechanisms in the case of DNA double stranded breaks (DSB). Mutations in *BRCA* genes compromise the HR pathway, leading to the reliance on non-homologous end joining (NHEJ) mechanisms for DNA repair. [50]. Findings indicate that the knockout of *PTEN*, a gene mutated in approximately 35% of GBM cases, reduced DNA repair protein RAD51 homolog 1 (RAD51) levels, leading to impaired HR. [28, 51] Intriguingly, it has been reported that inhibition of phosphate and tensin homolog deleted on chromosome 10 (PTEN) facilitates radio-sensitivity in GBM [52]. This demonstrated congruence between mechanisms regulating GBM resistance to radiotherapy in both genetic screens and in clinical settings.

#### ERCC4

In addition to emerging as a differentially expressed gene in Perturb-Seq screening analysis, *ERCC4* had previously been found to be differentially expressed in GBM in comparison to healthy tissues [53]. Additionally, *ERCC4* has been implicated in nucleotide excision repair (NER). This may underline the importance of NER in repairing bulky lesions induced during chemotherapy treatment of GBM [54].

#### LIG4

DNA ligase IV (LIG4), encoded by *LIG4*, deficiency has been suggested to potentiate significantly compromised NHEJ repair in response to damage resulting from ionizing radiation and associated sensitivity to DNA DSBs. [55] As mentioned, NHEJ is generally more error-prone than HR. In H7 GBM cells treated with TMZ and poly (ADP-ribose) polymerase (PARP) inhibitor, Ligase IV protein expression was observed to reduce cell viability [56]. Moreover, this finding, paired with evidence from genetic screening, suggests reliance on both NHEJ and HR mechanisms via Ligase IV in response to radiotherapy.

#### MRE11A

In GBM cell lines, the meiotic recombination 11 homolog A (Mre11a)- DNA repair protein RAD 50 (Rad50)- nibrin homolog (NBS1) (MRN) complex has been implicated in DNA DSB repair [57]. It has been shown that perturbation of retinoblastoma binding protein P4 (RBBP4), a histone binding protein, through RNAi, mediated sensitivity to radiotherapy in O6-methylC-DNA methyltransferase (MGMT)-depleted GMB. RBBP4 levels have been found to positively correlate with MRN levels. Moreover, lentiviral transduction of RBBP4 led to lower levels of phosphorylated ataxia-telangiectasia mutated (p-ATM) and p-serine threonine kinase Chk (p-Chk)2 in LN299 lines when compared to cells without exogenous RBBP4. However, in both cases, p-ATM and p-Chk2 levels were elevated in the presence of DNA damage compared to conditions without damage. This supports previous findings that in response to DNA DSBs, MRN drives increases in p-ATM levels. Importantly, Chk2 has been found to inversely correlate with M-phase inducer phosphatase 1 (Cdc25A) levels. It has been well established that high Cdc25A levels potentiate Cdk2 activity and subsequently drive S phase progression [58, 59]. As a result, high Chk2 levels resulting from lack of DNA repair may also impair proliferation. Importantly, this establishes a potential demand for adequate DNA repair mechanisms for GBM to maintain viability, and perhaps maintaining a proliferative phenotype, whilst undergoing infrared radiation (IR) damage. Interestingly, it has been observed that Mre11a may compete with MMR endo nuclease PMS2 (PMS2) for complexation with DNA MMR protein MLH1 (MLH1), thus negatively regulating MMR [60]. As will be discussed, MMR has been suggested to sensitize GBM to TMZ [40].

#### PRKDC

*PRKDC*, a gene which encodes DNA-dependent protein kinase (DNA-PK), has been shown to drive radio-resistance and chemoresistance in GBM [61, 62]. In osteosarcoma, it has been determined that DNA-PK catalytic subunit (PRKDC) recruits glycerophosphodiester phosphodiesterase 2 (GDE2) association with and stabilize guanine nucleotide-binding protein-G(S) subunit alpha (α) (GNAS). Stabilization of GNAS was found to activate the PI3K/AKT signaling pathway, which was hypothesized to contribute to chemotherapeutic resistance via AKT-mediated DNA damage response mechanisms [63]. Nonetheless, more work in GBM is needed to deconvolute mechanisms which may drive chemoresistance associated with *PRKDC*.

### Non-targeted CRISPR screen

For the purposes of this analysis, non-targeted CRISPR screens refer to genome-wide screens which do not impose external conditions which may bias genomic reads. One such screen has been performed on GBM cells using ten patient-derived GSC cell lines, with two fetal derived neural stem cells (NSC) lines as controls. 90 K TKOv1 or 70 K TKOv3 libraries were used to perform a CRISPR knock out (KO) screen. In this screen *SOX9*, *DOT1L*, *SOCS3*, *USP8*, and UFMylation pathway members were found to be integral to survival of GSCs in comparison to NSCs [64]. Additional studies have probed gene expression differences between three matched patient-derived GBM cultures of GSCs and differentiated GBM cells (DGCs) [65]. Following integration with published RNA-seq data, these two studies were cross analyzed and *YY1* was identified as a GSC-specific driver of GBM in comparison to both NSC and DGC [66]. *YY1* was also the only gene found to be differentially expressed between DGCs and GSCs and will be the focus of further discussion. In a different study, a CRISPR KO screening in NSCs and GSCs using the GeCKO library identified *PKMYT*_*1*_ as a gene critical, specifically, to GSC growth [67].

### Control of cell cycle and immortality

#### YY1

Studies have suggested that ying yang 1 (YY1) potentiates small ubiquitin like modifiers (SUMO)-specific protease 1 (SENP1) expression which was shown to repress SUMO modification of METTL3. It was observed that deSUMOylation of METTL3 promoted MYC expression. Importantly, MYC expression modulated by YY1 was implicated with driving GSC growth [68].

#### PKMYT_1_

Protein kinase membrane-associated tyrosine/threonine 1 (PKMYT_1_) phosphorylates CDK1 and thus prevents CDK1-cyclinB complexation. Furthermore, it has been demonstrated that *CDK1* downregulation via small interfering RNA (siRNA) significantly increased the *PKMYT*_*1*_ population of cells in the G2 phase of the cell cycle. These findings suggest perturbation of function can affect the G2/M phase transition [69, 70]. Functionally, this checkpoint may decrease DNA damage and subsequent apoptosis of GBM cells. In contrast to *CDK4* amplifications, *PKMYT*_*1*_ dependence demonstrates a need, some capacity, for regulated cell cycle in GBM to avoid excessive DNA damage accumulation.

### Biased genome-wide screens

Biased genome-wide CRISPR screens are defined as genetic screens which compare the impact of a perturbation in both the presence and absence of another perturbation. CRISPR KO screens were used to facilitate analysis of all DepMap dependencies determined E-twenty-six transcription (ETS) factors correlated with *TERT* expression in GBM in the presence of telomerase promoter mutations (TPM) [71]. Biased genome-wide screening was also performed using the Calabrese CRISPR activation (CRISPRa) system in tandem with scRNA -seq following induction of ferroptosis. The screen revealed that *LRP8*, encoding low-density lipoprotein receptor (LDLR)-related protein 8 (LRP8) was key to survival regardless of stress of ferroptosis. Additionally, an overlap in gene signatures between *GPX4* and *LRP8*-overexpressing cancer cells was found. Though initially established in *MYCN*-amplified neuroblastoma, these findings were later confirmed in the context of GBM [72, 73]. Elsewhere, in genome-wide CRISPR KO screening studies with the GeckOv2 library, E2F6 was upregulated in epidermal growth factor receptor (EGFR)-mutant U87 GBM cells expressing mutant EGFRvIII upon TMZ treatment [74].

### Mediation of metabolism

#### LRP8

*LRP8* modulates cancer ferroptosis by regulating selenocysteine levels, which are required for translation of the selenoprotein GPX4. Loss of *LRP8*, in turn, negatively affects GPX4 translation. Impairment in translation of GPX4 also promotes ferroptosis by impairing the removal of lipid peroxide accumulation. In turn, in GBM, *GPX4* and *LRP8* play a role in survivial [72, 75].

### Control of cell cycle and immortality

#### TERT

TPMs are genetic alterations which mediate cancer cell immortalization by activating telomerase expression. It has been shown, in melanoma, that population doublings plateaued in WT cells but continued linearly in TPM cells [76]. However, the role of these mutations has not yet been established in GBM.

#### E2F6

As mentioned, in EGFRvIII-mutant GBM treated with TMZ, E2F6 was shown to be capable of E2F1 inhibition; E2F6 knockout sensitized EGFRvIII GBM to TMZ. Additionally, it has been reported that heightened levels of nuclear factor kappa b (NFkB) upon TMZ administration in EGFRvIII-mutant GBM induced expression of E2F6 levels. During replication fork stalling, Chk1 was found to phosphorylate E2F6, thereby inhibiting E2F1 repression. Consequently, this inhibition of E2F1 by E2F6 led to stalling of cell cycle progression at the G1/S phase transition [74, 77]. E2F6 appears to play a critical role in regulating cell cycle progression and facilitating DNA repair during replicative stress in TMZ-resistant EGFRvIII-mutant GBM.

### Targeted CRISPR screen

Targeted CRISPR screens refer to methodologies which illicit perturbations in only a subset of the genome. Several targeted screening studies have been performed in GBM and related tumors. One such screen was performed with the purpose of testing eight different chemotherapy drugs in neuroblastoma cells. This was done using a CRISPR KO library composed of 655 druggable genes in 18 cell lines, ten of which were neuroblastoma lines, with the other eight being controls. From this screen, knockouts of *HDAC2*, *MET*, *KAEP1*, and *PRKDC* were found to demonstrate synergistically enhanced efficacy to when combined with various chemotherapy drugs [78]. In a different study, a CRISPR KO screen composed of 29,790 single-guide RNA (sgRNA)s was carried out to target 2,981 metabolic genes in a GMB43-patient derived xenograft (PDX) model. GBM cells were found to have the greatest dependence on *NDUFS8*, *SMPD1*, *COMTD1*, *CSE*, and *SMS* [79]. In a separate study, a CRISPR KO screen in the GBM U138 cell line was performed with a sgRNA library consisting of 4,574 genes which targeted cell motility and trafficking. This screen revealed *MAP4K4* to be the most pronounced regulator of GBM invasion [80]. Elsewhere, MacLeod et al. have observed enriched *MLH1* and *MSH2* sgRNA abundance in response to high doses of TMZ in a positive selection CRIPSR KO screen. On the other hand, disruption of *ZC3H7A*, *MCM8* or *MCM9* were identified to drive GSC sensitivity to TMZ following a negative selection CRISPR KO screen in response to sublethal doses of TMZ [64].

### Mediation of metabolism

#### KEAP1

Loss of kelch-like ECH-associated protein 1 (KEAP1) leads to release of Nrf2, allowing its translocation to the nucleus, where it activates antioxidant gene transcription, helping cells counteract oxidative damage [81]. As a separate response to oxidative stress, studies have suggested that EGFR-mediated NADPH oxidase (NOX) assembly facilitates ROS generation in GBM [82, 83]. Heightened expression of genes associated with NOX assembly has been found to increase glioma cell proliferation [84]. The emergence of *KEAP1* as a genetic regulator in chemotherapy-treated tumors may benefit from further investigation owing to this gene’s established role in antioxidative damage control.

#### NDUFS8

*NDUFS8* has been found to play a critical role in the function of mitochondrial complex I. Silencing of this gene has been shown to result in elevated ROS, reduced oxygen consumption, and enhanced lipid peroxidation [85].

#### SMPD1

Sphingomyelin phosphodiesterase 1 (SMPD1) has been found to serve as a mediator in the reversible conversion of sphingomyelin to ceramide in GBM39. Furthermore, in GBM, data have suggested that SPMD1 depletion impairs EGFR signaling and reduces cell viability. Studies have also shown that in GBM39 cells treated with shSPMD1 to deplete SPMD1, those with constitutively active AKT exhibited higher viability compared to SPMD1-depleted GBM39 cells. This further underlines the importance of SMPD1 in regulating the EGFR axis [86].

#### COMTD1

There currently exists sparse literature surrounding *COMTD1* in context of GBM. In a study, GBM cells lacking Annexin 2 exhibited significantly elevated levels of catechol O-methyltransferase domain-containing protein 1 (CMTD1) relative to cells expressing canonical Annexin 2. Additionally, evidence suggests that Annexin 2, a cytoskeletal protein, is overexpressed in certain GBM subsets, which are generally associated with a poorer prognosis [87]. This may suggest compensation with *COMTD1* occurs in cases of Annexin 2 deficiency, however, further studies should be done. In other tumors, such as melanoma, it has been documented that knock-out of this enzyme was responsible for elevated GSH disulfide (GSSG) levels in B16F10 cells [88].

#### CSE

*CSE* has been documented to encode cystathionine gamma (γ)-lysate (CTH), which was identified as an antioxidant buffer playing a role in mitigating damage caused by ROS. Mechanistically, CTH was shown to convert cystathionine to cysteine. A similar mechanism for cysteine has been demonstrated in mouse neuronal cells [89], where the expression of CTH was shown to be linked with higher GBM invasion. Similarly, elevated H_2_O_2_ levels have been reported to enhance GBM invasion. Moreover, studies indicate that CTH-deficient GBM cells exhibit lower viability when exposed to H_2_O_2_ when compared to controls. These findings highlight the critical role of CTH in mitigating ROS-induced cellular damage during invasion [79]. Elsewhere, cysteine deprivation, an upstream component of GSH synthesis, has been shown to lead to both depleted GSH and ferroptosis. Blockade of GSH did not, however, induce ferroptosis, which indicates that cysteine may act independently of GSH to reduce lipid ROS. These findings may underline the importance of cystine production via CTH in prevention of ROS and ferroptosis [90]. Related to this, in low glucose conditions, U251 cells were found to exhibit significant cell death in media containing cysteine. Additional studies have supported that in low glucose conditions, treatment of cancer cells with N-acetyl cysteine (NAC), lower NADP + /NADPH and GSH/GSSG ratios were observed, along with increased mitochondrial H_2_O_2_ [91, 92]. Together, these findings suggest a need for regulation of cysteine levels in maintaining cell viability under various environmental contexts, further emphasizing the importance of CTH.

#### SMS

*SMS* encodes spermine synthase (SMS) and catalyzes the conversion of spermidine (SPD), a naturally occurring polyamine, to spermine. GBM has been reported to have higher levels of SPD, which were shown to drive tumor aggressiveness [93].

### Control of cell cycle and immortality

#### HDAC2

The role of histone deacetylase 2 (HDAC2) in GBM appears to regulate acetylation of histones 3 and 4 which were in turn found to potentiate brain tumor stem cell (BTSC) self-renewal. Data have suggested this occurred through modulation of transforming growth factor beta (TGF-β) pathway proteins, specifically mothers against decapentaplegic homolog 3 (Smad3). Knockout of Smad3 was shown to facilitate reduced stem-like properties in GBM BTSC cell line BT67 [94].

#### MAP4K4

The mechanism driving mitogen-activated protein kinase 4 (MAP4K4)-dependent motility is not well established in GBM. It has been observed in ovarian cancer, however, that MAP4K4 mediates phosphorylation of A disintegrin and metalloprotease 10 (ADAM10) which prevents N-Cadherin degradation, thereby promoting motility and metastasis [95]. Importantly, cell cycle arrest at G2/M transition was found upon MAP4K4 inhibition in pancreatic cancer cell lines [96].

### DNA repair and damage response

#### MET

Phosphorylation of ATM and, correspondingly, the phosphorylation of Chk2 and RAD51 following irradiation were shown to decrease upon mesenchymal-epithelial transcription factor (MET) inhibition in GBM. This finding suggests that MET is involved in tumor progression through DNA damage response [97].

#### ZC3H7A

The role of *ZC3H7A* in GBM has not been well characterized beyond identification in primary CRISPR screens. In functional studies, it was determined that zinc finger CCCH-type containing 7 A (ZC3H7A) regulates intracellular stress management mechanisms [64]. However, more work characterizing specific mechanistic interactions may provide a molecular basis for viability of ZC3H7A inhibition in TMZ-resistant GBM.

#### MCM8/MCM9

Minichromosome maintnece (MCM)8 and MCM9, proteins which associate to form a helicase complex, have been identified as essential for MCM8 interacting protein (IP)-dependent HR. Interestingly, mutation in MCM8IP was shown to lead to significant reduction in HR by preventing association with MCM8 and MCM9 [98]. It follows that MCM8 and MCM9 may play important roles in facilitating DNA repair in GBM under chemotherapeutic treatment.

#### MLH1/MSH2

In EGFRvIII-mutant GBM, it has been shown that *MGMT* promoter methylation was associated with sensitivity to TMZ. Moreover, TMZ-mediated O6-methyl (O^6^-Me) guanine damage was shown to trigger an elevation in MMR, mediated by MLH1 and DNA MMR protein MSH2 (MSH2). In absence of MGMT-mediated demethylation in response to O^6^-Me guanine, MMR was shown to proceed in a futile cycle where thymidine mis-paired to methylguanine is continually excised and replaced. These findings agree with other studies which revealed that depleted MLH1 and MSH2 levels may drive TMZ resistance in GBM [99, 100].

## RNAi screens

In contrast to studies previously discussed, RNAi screens refer to those where siRNA is introduced to cells to induce post-transcriptional knockdown of translated genes. This type of screen differs from CRISPR screens which generally intend to limit transcription of DNA at specific sites. Only one RNAi screen will be discussed in this review. In this study, an siRNA library of deubiquitylating enzymes (DUBs), expressed in HEK293 cells, was used to perform the screen in U87, U251, T98G, and LN229 GBM cell lines. Further attenuation of mesenchymal (MES) GSC tumorgenicity was established conditionally upon ubiquitin-specific peptidase 21 (USP21) knockdown [101].

### Mediation of metabolism

#### USP21

USP21 has been established as a mediator of Forkhead box D1 (FOXD1) ubiquitination reversal. Moreover, polyubiquitination of FOXD1 was reported to be conducive to degradation of the protein. FOXD1 depletion following USP21 knockout led to a concurrent decrease in p-signal transducer and activator of transcription (p-STAT3) levels. In HAP-1 cells, ATP production and subsequent cell proliferation were correlated with depleted p-STAT3 following USP21 knockout [101, 102]. Further work, specifically in GBM, may probe the mechanisms behind USP21-mediated translocation of STAT3 to the mitochondria. Nonetheless, the STAT3/FOXD1 axis remains a potential contributor to GBM pathology. Notably, studies indicate that FOXD1 drives the MES phenotype in GBM cells, while USP21 is required to maintain stable FOXD1 expression [101]. Furthermore, MES GBM cells have demonstrated enrichment in clusters which were associated with both high glycolytic activity and lactate accumulation [103].

### Two cell type (TCT) screens in GBM

Unique to previously discussed genetic screens, TCT screens in GBM refers to cases where effector cells are co-cultured with GBM cells transduced with sgRNA libraries. In a TCT CRISPR KO screen in GBM which leveraged the Brunello sgRNA library, *CHMP2A* KO in GSCs was shown to drive sensitivity to killing by NK cells. Further investigation also determined that *CHMP2A* KO concurrently increased NFkB and, although only shown in Cal27 cells, decreased soluble TNF-related apoptosis-inducing ligand (TRAIL) and MHC class 1 chain-related protein (MIC)A and MICB levels [104].

### Control of cell cycle and immortality

#### CHMP2A

NFkB inhibition by Bay 11-7082 was found to lead to increased apoptosis of GBM cells following TMZ treatment. This was proposed to be driven through increased Bax and concurrently decreased B-cell lymphoma/leukemia type 2 protein (Bcl-2) levels following inhibition of NFkB in addition to TMZ treatment [105]. Together, these findings suggest that NFkB may play an anti-apoptotic role in GBM. This is in agreement with previously mentioned studies which had found that high NFkB regulated elevated E2F6 levels in response to TMZ, thus helping maintain GBM viability [74].

### Genetic screens in NK cells

#### TCT Screens in NK cells

In one TCT screen in NK cells, sgRNA libraries were transduced into mouse primary NK (pNK) cells. Subsequently, transduced pNK cells were injected into syngeneic mice bearing GL261 tumors. Genomic DNA was analyzed 7 days after NK cell administration. In parallel, scRNA-seq was performed on NK cells isolated from treated mice. These findings have identified *CALHM2* as a convergent hit between the two parallel screens. Specifically, *CALHM2* sgRNA had been reportedly enriched in tumor infiltrating NK cells. Within the same study, scRNA-seq revealed 49 upregulated and 211 downregulated genes in *CALHM2*-KO cells. In the same study, *CALHM2*-KO NK cells were shown to have heightened cytotoxicity [106]. In a different CRISPR KO screen, NK cells derived from cord-blood (CB) were initially transduced with a library targeting transcription factors and placed in co-culture with Panc1 or PATC148 tumor cells. *IL2RG* and *JAK3*, a member of the JAK/STAT signaling pathway, were identified as necessary for maintaining NK cell functionality. In contrast, sgRNAs that were positively enriched identified tumor protein p53 (TP53) as a potential therapeutic target; its knockout in NK cells may enhance their anti-tumor activity. In the same study, NK cells derived from CB were transduced with a genome-wide CRISPR library, then, first challenged with Capan-1 cells and, in separate experiments, with TGF-β, lactic acid, or hypoxia. Through these two genome-wide screens and integration with CRISPR knockout datasets from T cells, *MED12, CCNC*, and *ARIH2* were identified as candidate genes whose knockout enhances the anti-tumor response of NK cells. However, in an orthotopic mouse model implanted with PATC148 tumors, only *CCNC* and *ARIH2* double knockout CAR-NK cells—not *MED12*-knockout CAR-NK cells—were able to significantly reduce tumor burden. [107].

## NK cell implications

**Fig. 2:**
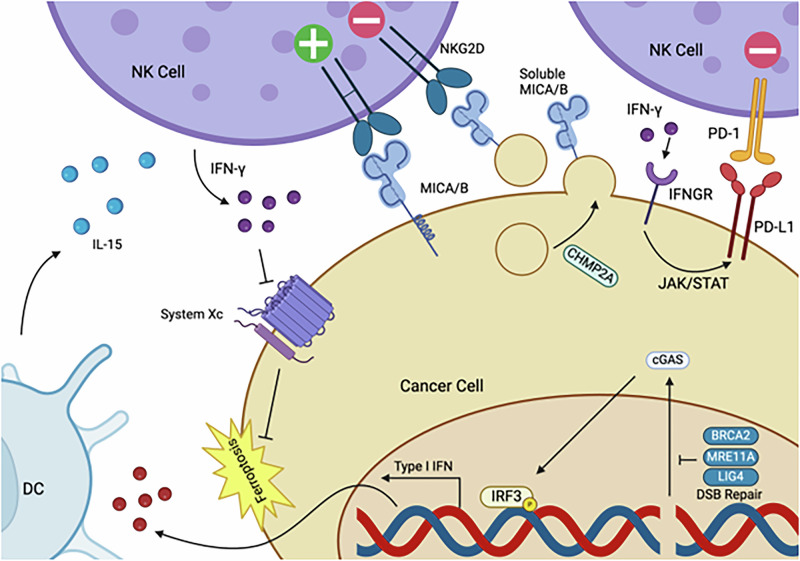
NK cell activation is modulated by genes identified in GBM cells through genetic screens. DNA damage in cancer cells in the form of DSBs drives IRF3 phosphorylation mediated through the cGAS pathway, which can be inhibited by competent DSB repair mechanisms. These mechanisms can involve BRCA2, MRE11A, and LIG4. Phosphorylated IRF3 drives production of Type I IFN molecules [55, 57, 154, 155, 174]. These type I IFN molecules prompt IL-15 secretion by DCs, which signals NK cells to secrete IFN-γ [156, 157]. Secretion of IFN-γ represses system x_c_ expression on cancer cells, indirectly promoting cancer cell death through ferroptosis [165–167, 181]. Soluble IFN-γ, secreted by NK cells, also binds IFNGR on cancer cells which drives PD-L1 expression dependent on the JAK/STAT pathway. PD-L1 on tumor cells is inhibitory to NK cells following binding to PD-1 [148, 182, 183]. Soluble MICA/B is inhibitory to NK cells following binding with NKG2D, and its levels increase with the release of exosomes from cancer cells, a process mediated in part by CHMP2A. In contrast, surface expression of MICA/B on cancer cells activates NK cells through binding to NKG2D expressed on the NK cell surface [104, 193].

Each of the genes identified through various genetic screening methodologies—OCT perturb-seq, non-targeted CRISPR, biased genome wide, targeted CRISPR, RNAi, TCT screens in GBM, and TCT screens in NK cells—in proceeding discussion will be described to play a role in DNA repair and damage response, control of cell cycle and immunity, or mediation of metabolism in some capacity (Table 1). In addition, many of these genes have been found to have an immunomodulatory impact on NK cells, by either enabling cancer resistance to NK cell killing, by being critical to NK cell survival and effector responses (Table 2 and Fig. 2). In this review we will also define and reclassify these genes based on their known impact on NK cell function- either in modulating cell surface receptor signaling or soluble factors in the TME (Table 3).

**Table 2:** Genes impacting NK cell function identified from genetic screens involving NK cells.

Gene	Screen type	Cell type target of screen	CRISPR approach	Reference
***CHMP2A***	TCT	**GBM**	CRISPR KO	[104]
***CALHM2***	TCT	**NK**	CRISPR KO	[106]
***ARIH2***	TCT	**NK**	CRISPR KO	[107]
***CCNC***	TCT	**NK**	CRISPR KO	[107]

**Table 3:** Functional assignment of genes identified from genetic screens.

Function associated with various genetic components	Mediation of metabolism	Control of cell cycle	DNA repair and damage response	Cell surface receptor signaling between NK cells and GBM	Soluble factors in the TME regulating NK cell response to GBM
**Name of Gene**	*CYP19A1*	*BORA*	*BRCA2*	*CYP19A1*	*BORA*
	*HSD17B10*	*YY1*	*ERCC4*	*ERCC4*	*BRCA2*
	*LRP8*	*PKMYT*_*1*_	*LIG4*	*LIG4*	*YY1*
	*KEAP1*	*TERT*	*MRE11A*	*MRE11A*	*TERT*
	*NDUFS8*	*E2F6*	*PRKDC*	*PRKDC*	*KEAP1*
	*SMPD1*	*HDAC2*	*MET*	*YY1*	*SMPD1*
	*COMTD1*	*MAP4K4*	*ZC3H7A*	*PKMYT1*	*CSE*
	*CSE*	*CHMP2A*	*MCM8*	*LRP8*	*HDAC2*
	*SMS*		*MCM9*	*E2F6*	*MET*
	*USP21*		*MLH1*	*HSD17B10*	*ZC3H7A1*
			*MSH2*	*NDUFS8*	*MLH1*
				*COMTD1*	*USP21*
				*SMS*	*CHMP2A*
				*MAP4K4*	*ARIH2*
				*MCM8*	*CCNC*
				*MCM9*	
				*CALHM2*	

### Cell surface receptor signaling between NK cells and GBM

Adaptive immune cells, such as T-cells, generate a diverse array of T-cell receptors (TCRs) through genetic recombination to recognize potentially foreign cells. TCRs allow T-cells to identify foreign cells through peptide signatures bound to major histocompatibility complex (MHC) class I receptors [108, 109]. NK cells express germline-encoded activating and inhibitory receptors. Among these, NK cells express inhibitory receptors, such as cluster of differentiation (CD)94/NKG2-A/NKG2-B type II integral membrane protein (NKG2A) when binding MHC class I receptors. Regardless of variability in peptide binding pockets between classical MHC class I encoded by *HLA-A*, *HLA-B*, or *HLA-C* or *HLA-E*, presentation on target cells can inhibit effector function [110–112]. In addition to MHC class I recognition, NK cells are endowed with a variety of other activating and inhibitory receptors. These receptors enable recognition of potentially cancerous cells; the balance of these activating and inhibitory receptor interactions drives NK cell activation [113–115]. Further, upon commitment to NK cell activation, synapse formation and granzyme release facilitate cancer cell lysis [116]. Attempts to block inhibitory signals, and potentially trigger effector function through Fc recognition, have been pursued through immune checkpoint inhibitor (ICI) strategies [117–119]. Although TME settings modulate the expression of receptors on NK cells, other therapeutics have been shown to have the capacity to induce receptor expression changes. For instance, a variety of chemotherapeutic drugs have been found to upregulate programmed death-ligand 1 (PD-L1) and bolster efficacy of combined chemotherapy and immunotherapy approaches [120]. Additionally, cell surface receptor expression may vary from patient to patient, even when diagnosed with the same cancer type [121]. As a result, alterations in genes identified in GBM genetic screens demonstrate immunomodulatory effects resulting from alterations in the cell surface receptor landscape expressed on cancer cells. Specific alterations discussed potentiate differential responses to ICI due to upregulation of PD-L1 and other inhibitory ligands. The following genes have emerged as hits from OCT perturb-seq, non-targeted CRISPR, biased genome wide, targeted CRISPR, and TCT screens in NK cells. These genes have been shown to modulate the cell surface receptor interactions between NK cells and cancer cells.

#### CYP19A1

*CYP19A1* was found to impact response to radiotherapy in an OCT perturb-seq screen in GBM [40]. In both HT29 and HCT116 cell lines, inhibition of CYP19A1 was found to decrease TGF-β levels. It is worth noting that inhibiting CYP19A1 also upregulated PD-L1 expression [122]. Importantly, TGF-β targeting was found to restore NK cell metabolism and generally restore innate immunity [123]. Specifically, TGF-β was found to negatively correlate with activating receptor NKG2-D type II integral membrane protein (NKG2D) on NK cells [124]. In addition, the *CYP19A1* oncogene was found to be implicated in NK cell effector function.

#### ERCC4

Radiotherapy response of GBM cells was seen in an OCT perturb-seq screen to depend, in part, on *ERCC4* [40]. The excision repair cross-complementing (ERCC) family of proteins has been associated with NER. Moreover, variable differential expression of *ERCC1-6* has been observed between cancer types [125]. In pan-cancer patients with *ERCC* mutations, including *ERCC4*, overall survival (OS) was greater than that for cohorts without *ERCC* mutations. Moreso, enhanced survival of the *ERCC* mutant cohort in response to ICI administration was observed in comparison with non-mutant *ERCC* cohorts. Furthermore, it was determined that tumor neoantigen burden was significantly enhanced in *ERCC*-mutant cancer and a significant increase in infiltrating CD8 T cells. While there was not a significant increase in NK cell infiltration, enhanced efficacy of ICI potentially signifies general importance of immune response to *ERCC*-mutant cancer [126].

#### LIG4

In an OCT perturb-seq screen findings have suggested *LIG4* deficiency sensitizes GBM to radiotherapy [40]. *LIG4*, which encodes LIG4, associates with x-ray cross complementing protein 4 (XRCC4) [127]. *XRCC4* has been positively associated with both NK cell signatures and immune checkpoint expression. Specifically, in GBM, significant positive correlation was found between *XRCC4* and *BTLA* [128]. B- and T-lymphocyte attenuator (BTLA) has been shown to suppress NK cell function when agonized, and potentially drive cancer suppression upon BTLA blockade [129]. Although *XRCC4* has been suggested to play a role in modulating the immune system, direct experimental evidence on the interaction between cancer, with differential *XRCC4* expression, and specific immune cell types, is lacking.

#### MRE11A

Mre11a was found, in an OCT perturb-seq screen, to modulate GBM response to radiotherapy [40]. Not much is known about the immunomodulatory impact of Mre11a deficiency in cancer. RBBP4, known to modulate MRN, a complex containing Mre11a, has been found to negatively correlate with both CD56^dim^ and CD56^bright^ NK cell infiltration in non-small cell lung cancer (NSCLC) [130]. Studies have also shown a correlation between RBBP4 and immune cell infiltration in low grade glioma (LGG). However, additional studies are needed to define RBBP4-dependent alterations within the GBM TME [131].

#### PRKDC

*PRKDC* has emerged as a radiotherapy resistance driver gene in GBM from a OCT perturb-seq screen, and a driver of chemotherapy resistance in neuroblastoma in a targeted CRISPR screen [40, 78]. In multivariate analyses, *PRKDC* mutations were identified as being positively associated with improved survival in patients receiving treatment with ICIs. In *PRKDC*-deficient tumors, there were significantly greater messenger RNA (mRNA) expression of NK-related genes *KLRC1* and *NCR1* which encode NKG2A and NK cell p46 related protein (NKp46), respectively [132]. NKG2A is a well-established inhibitory receptor expressed on a majority of NK cells, while NKp46 serves as an NK cell activating receptor [133]. This suggests that increased efficacy of ICI therapy may be bolstered by an enhanced population of immune effector cells.

#### YY1

*YY1* has been identified as a GSC-specific dependency in a CRISPR screen, serving as a genetic driver of GBM. [66] As previously stated, increased levels of YY1 were determined to drive heightened stability of MYC mRNA. Data suggests in absence of c-MYC, MHC class 1 chain-related protein MICB, UL16 binding protein (ULBP)1, and ULBP2 surface levels were significantly higher than WT in K562 cells [134]. While this finding was not shown in the context of GBM, this evidence suggests YY1 plays a role in reducing expression of cognate ligands on target cells for NK cell activating ligands.

#### PKMYT1

In a non-targeted CRISPR Screen, the gene *PKMYT*_*1*_ was seen to drive GSC growth [67]. Significantly higher levels of activated NK cells were observed in the TME of tumors with high PKMYT1 expression over tumors with low PKMYT1 expression [135]. It is worth noting that these findings merely identified PKMYT1 as a marker associated with immunomodulation, with much on its functional effects not being known.

#### LRP8

As discussed, in a biased genome wide screen, selenoprotein p (SeP) receptor LRP8 overexpression has been found to drive GBM resistance to ferroptosis [72]. The impact of LRP8 expression in cancer and the effects on NK cells are only partially known. Studies have suggested that SeP levels positively correlate with six immune cell types; none of which, however, were NK cells. It was discussed that SeP uptake has been found to be mediated by LRP8 [136]. Additionally, as previously mentioned, elevated *LRP8* levels was associated with resistance to GPX4 inhibition [72]. GPX levels have also been shown to associate with NK cell viability in the TME [137]. Discovery of the inhibitor NF611 selectively abolished GPX4 levels in cancer cells without impacting GPX4 levels in T cells or NK cells. It was found that CD8 + T cells were responsible for driving a significant reduction in tumor volume upon treatment with NF611 upon CD8 blockade in vitro and in *Rag1*^-/-^ mice in vivo. Further analysis on the response in vivo is lacking, however, tumor clearance has been associated with decreases in GPX4 levels within cancer cells [138]. Generally, this evidence demonstrates the potential impact of LRP8 on the expression of GPX4 in cancer and immune cells.

#### E2F6

As previously mentioned, E2F6 emerged from a biased genome-wide screen in EGFRvIII GBM [74]. There exists an apparent paradoxical relationship between knockout of E2F6 and the associated immune response. E2F6 expression was shown to negatively correlate with immunomodulatory genes in GBM. Specifically, expression of the gene *ICOS* was significantly negatively correlated with E2F6 expression, whereas U87 cell growth was reduced upon E2F6 knockout through short hairpin RNA (shRNA) [139]. Importantly, Inducible T-cell COStimulator (ICOS) deficiency has been associated with NK cell depletion [140]. Nevertheless, more work into the role of E2F6 loss in GBM may further elucidate the immune impact of this oncogenic driver gene.

#### HSD17B10 & NDUFS8

The gene *HSD17B10* emerged as a modulator of response to radiotherapy in a OCT perturb-seq screen in GBM. Whereas *NDUFS8* was implicated as a metabolic driver gene in GBM in a targeted CRISPR screen [40, 79]. Upon treatment with combined tyrosine kinase inhibitor (TKI) and anti-PD-L1 antibody, expression of HSD17B10 was found to negatively correlate with prognosis; lower expression of HSD17B10 best corresponded with patient response. Additionally, knockout of nicotinamide adenine dinucleotide phosphate (NADH) dehydrogenase ubiquinone 1 β subcomplex (NDUFB)8 in combination with ICI led to slower tumor growth compared to WT NDUFB8 groups treated with anti-PD-L1. Furthermore, levels of oxidative phosphorylation (OXPHOS)-related proteins HSD17B10 and NDUFB8 prior to ICI therapy were found to follow a similar trend in predicting patient outcome. Along similar lines, the gene *NDUFS8*, encoding NADH:ubiquinone oxidoreductase core subunit (NDUFS)8, which, similarly to NDUFB8, is a component of NADH:ubiquinone oxidoreductase, has been closely implicated in driving patient outcome. It was further determined that expression of OXPHOS genes positively correlated with hypoxia levels [85, 141, 142]. Importantly, high levels of hypoxia have frequently been associated with suppressed NK cell function, although oxygen levels differentially affect NK cells [143]. Nevertheless, *HSD17B10* and *NDUFS8* are closely implicated in OXPHOS-dependent response to ICI.

#### COMTD1

In a targeted CRISPR screen, the gene *COMTD1* was suggested to be a genetic, metabolic dependency in GBM cells [79]. Evidence surrounding immune cell infiltration and the general landscape of the GBM TME in *COMTD1*-deficient cancer is sparse. In other tumors such as breast cancer, *COMTD1* was shown to be upregulated in high-risk patients. Although data has shown that there are fewer CD56dim NK cells in low-risk breast cancer compared to high-risk breast cancer, both CD8 T cells and CD56^bright^ NK cells were significantly elevated in low-risk samples. In addition, *CD276*, the gene encoding B7 homolog 3 (B7-H3), an inhibitory ligand overexpressed in certain cancers, was elevated in the high-risk group. It is worth noting, however, that adenosine receptor A2A (A2AR) was elevated in low-risk groups when compared to high-risk groups. Importantly, A2AR has been found to be associated with high grade breast cancer when expressed on cancerous tissue and immunosuppressive when expressed on NK cells [144–146]. Further work individually probing the impact of *COMT1* in GBM on A2AR levels in NK cells may help elucidate these seeming conflicting findings. Nevertheless, *COMTD1* has been associated with high-risk cancer patients that generally exhibit lower immune effector tumor infiltration.

#### SMS

In a targeted CRISPR screen in GBM, *SMS* was identified as a metabolic dependency [79]. Spermine was shown to drive PD-L1 expression through β-catenin phosphorylation mediated by p-Akt. Akt phosphorylation is governed by calcium-sensing receptor (CaSR), which was significantly associated with PD-L1 mRNA levels following treatment with spermine [147]. Expression of PD-L1 was shown to be capable of NK suppression [148]. While genetic screening identified *SMS* and not specifically spermine, the control of both SPD and spermine by SMS may play a role in both immunosuppression and cancer aggressiveness.

#### MAP4K4

As discussed, *MAP4K4*, a GBM motility gene, was identified in a targeted CRISPR screen to drive GBM invasion [80]. Studies have suggested that MAP4K4 mediates stable expression of N-Cadherin. Furthermore, N-Cadherin was found to associate with killer cell lectin-like receptor subfamily G member 1 (KLRG1), an NK surface receptor, thus driving NK exhaustion and immune evasion in tumors [149].

#### MCM8/MCM9

Emerging from a targeted CRISPR screen, *MCM8* and *MCM9* knockout were found to drive GBM sensitivity to TMZ [64]. Presently, evidence underlying the mechanistic features governing NK cell function and presence in cancers with high expression of MCM8 or MCM9 is not abundant. However, findings suggest that in hepatocellular carcinoma (HCC) tissue samples with high MCM8 expression, a lower presence of activated NK cells was detected [150]. This suggests that in addition to driving resistant GBM, *MCM8* may play a role in suppressing activated NK cell infiltrating into tumor tissues.

#### CALHM2

As mentioned, KO of *CALHM2* has been found to be correlated with NK cell infiltration in GBM following a TCT screen in NK cells [106]. Additionally, genes associated with enhanced Wnt pathway signaling and reduced translational regulation were observed in *CALHM2* KO NK cells. There is sparse evidence correlating increased Wnt signaling genes in NK cells with cytotoxicity of GBM. It was reported that decreased the protein poly(A) binding protein cytoplasmic1 (PABPC1) led to decreased p-STAT-3 levels; the gene encoding PABPC1 was downregulated upon *CALHM2* KO [151]. Importantly, decreased STAT-3 levels in NK cells were shown to enhance tumor surveillance by upregulating DNAX accessory molecule-1 (DNAM-1) expression. DNAM-1 has been reported to be an activating receptor on NK cells [152].

### Soluble factors in the TME regulating NK cell interaction with GBM

A number of soluble factors in the TME function to suppress immune cells while potentiating establishment and progression of solid tumors [153]. Specifically, genomic instability characteristic of many cancers including GBM elicit engagement of the cyclic guanosine monophosphate (GMP) adenosine monophosphate (AMP) synthase (cGAS)- stimulator of IFN genes (STING) pathway which prompts secretion of type one interferon (IFN) into the TME [154, 155]. As a result, type one IFN presence prompts interleukin (IL)-15 secretion by dendritic cells (DC)s which plays a role in NK cell activation; the resulting activated NK cells demonstrate capacity for IFN-γ secretion [156, 157]. Accordingly, IL-15 has been shown to be critical in enabling long term persistence of NK cell populations and anti-tumor cytotoxicity without initiating graft-versus-host disorder (GvHD) in allogenic adoptive transfer studies [158–160]. Furthermore, elevated IFN-γ in the TME drives upregulation of PD-L1 on tumor cell surface [161, 162]. Nevertheless, combination of type one IFN and anti-PD-L1 therapy has demonstrated efficacy in clinical trials [163]. However, type one IFN signaling in the TME is also associated with immunosuppression, such as heightened regulatory T cell (Treg) recruitment to the tumor [164]. IFN-γ levels in the TME also downregulate SLC7A11, a key protein involved in the system x_c_ complex. Importantly, both the system x_c_^-^ -GSH-GPX4 axis and Nrf2 mediated regulation of antioxidant proteins work to control elevated ROS associated with the TME [165–167]. It should be noted that soluble factors MICA/B, when cleaved by proteases on cancer cells as well as TGF-β, suppress NK cell secretion of IFN-γ [168–170]. Conversely, soluble chemokines released into the TME drive NK cell homing to the site of the tumor [171]. The following genes have been identified through one-cell-type perturb seq, non-targeted CRISPR, biased genome wide, targeted CRISPR, RNAi, TCT screens in GBM, and TCT screens in NK cells. These genes have documented, established implications on concentrations of soluble factors, thus affecting the capacity to affect immunosurveillance of tumors.

#### BORA

Deficiency of *BORA*, identified in an OCT perturb-seq screen, was shown to sensitize GBM to radiotherapy [40]. Literature surrounding the impact of *BORA* expression in cancer on the host immune system is sparse. However, the impact of Aurora A, a protein kinase activated by BORA, on playing an immunosuppressive role in the context of cancer is better established. Specifically, findings have suggested that Aurora A inhibition downregulates p-STAT3, correlates to lower ROS levels in the TME and higher immune activation [172]. Importantly, ROS has been found to negatively correlate with NK cell infiltration in cancer tissues [173]. Together, *BORA* may not only play an important role in cancer cell viability in response to radiotherapy but also increase immune suppression through regulating ROS levels in the TME.

#### BRCA2

In an OCT perturb-seq screen in GBM, *BRCA2* knockout was found to sensitize GBM to radiotherapy [40]. It had been established that prolonged deficiency of *BRCA2* induces phosphorylation of IFN regulatory factor 3 (IRF3) and STAT1. Higher levels of cGAS micronuclei were observed in *BRCA2*-deficient cells, while cGAS-STING was considered responsible for IRF3 phosphorylation. Moreover, IRF3-mediated production of type I IFN was suggested to be the driving force for the observed STAT1 phosphorylation and expression of innate immune response genes such as *IFIT1* in breast cancer type 2 susceptibility protein (BRCA2)-deficient H1299 cells. These findings indicate *BRCA2* deficiency is responsible for triggering increased expression of innate immunity genes [174]. Furthermore, type I IFN, namely IFN-α, has been shown to drive STAT1 phosphorylation and promote NK cell cytotoxicity [175]. Although *BRCA2* deficiency has been thought to drive mutations in cancer, these findings may suggest increased type I IFN in *BRCA2*-deficient cancer may stimulate NK cell killing of cancer cells.

#### YY1

Perturbation of *YY1* was found, in a non-targeted CRISPR screen, to lead to increases in IFN-β levels in GSCs [66]. Moreover, studies in GL26-GAL1 knockdown cells have suggested that IFN-β association with IFN α/β receptor 1 (IFNAR1), a surface ligand on NK cells, triggers GBM killing. It was suggested that exosomes released from glioma cells expressing miR-1983 mediated toll like receptor (TLR)7-dependent release of IFN-β from DCs, triggering NK cell killing of GBM cells [176]. These findings suggest a circuit, in NK cells, which facilitates killing of GBM cells in response to IFN-β.

#### TERT

As described, findings have implicated *TERT* promoter mutations as drivers of GBM, identified through biased genome-wide screens [71]. In response to telomerase inhibitor 6TdG, NK-92 cells in co-culture with H510 cells promoted an increase in cancer cell killing by nearly 20% in comparison to untreated co-cultures. Importantly, there was significant difference in 6TdG in metastatic models between immunocompetent mice and NK-depleted mice. Further investigation revealed that due to accumulation of DNA damage upon 6TdG administration, activation of the cGAS-STING pathway facilitated effector function through type I IFN [177]. These findings agree with genome-wide screening outcomes and further highlight the potential importance of NK effector function for in vivo telomerase inhibitor efficacy.

#### KEAP1

In a targeted CRISPR screen in neuroblastoma cells, *KEAP1* knockout was shown to enhance efficacy of chemotherapy [78]. Recall Nrf2 release was observed in cases of KEAP1 deficiency [81]. Enhanced Nrf2 levels in NK cells induced elevated lysis of K562 in environments with heightened levels of ROS [178]. Similarly, elevated Nrf2 levels were responsible for increased GBM proliferation. Taken together, Nrf2 has a potential role in the maintenance of NK cell cytolytic capacity in high ROS environments.

#### SMPD1

The gene *SMPD1*, which has been identified as a metabolic driver of GBM through a targeted CRISPR screen, encodes the protein SMPD1 [79]. *SMPD1* deficiency has been implicated in cell cycle arrest in A549 cells and decreased cell counts in H520 populations. In serum starvation conditions, increased apoptosis of A549 cells deficient in *SMPD1* over that of *SMPD1*-expressing cells was observed. These findings have suggested that although deficient *SMPD1* expression may drive apoptosis in low nutrient conditions, cancer cells with competent *SMPD1* levels maintain higher levels of proliferation regardless of access to nutrients. In contrast, findings have suggested that in M38 tumor-bearing mice, *SMPD1*-deficient CD8 T cells demonstrated lower apoptosis and increased *Grzb* level expression [179]. While there is limited work exploring NK cell function in *SMPD1*-deficient cancers, studies have suggested that upon SMPD1 knockdown, NK cell effector function was significantly enhanced. In such settings, morphological changes to NK cell membrane depended on sphingomyelin [180]. Together, these findings suggest that *SMPD1* may deplete sphingomyelin in the TME, thus suppressing NK cell function.

#### CSE

*CSE*, identified as a metabolic driver gene in GBM through a targeted CRISPR screen, has been shown to promote intracellular cysteine synthesis [79]. Cysteine starvation was shown to deplete GSH levels, leading to ferroptosis [90]. Furthermore, findings have suggested a negative correlation between IFN-γ and SLC7A11, a cystine transporter which mediates lipid peroxidation and ferroptosis. Additionally, cystinease was found to significantly reduce cancer cell viability in response to IFN-γ. Findings have supported that CD8 T cells have the capacity to induce higher lipid ROS in cancer cells. Moreover, the same study showed that CD8 T cells expressing high levels of IFN-γ were associated with lower tumor flux [181]. While there exists limited information on *CSE* in an immune context, cystine levels have been reported to promote cancer cell defense against ferroptosis. Together, these findings support a mechanism in which IFN-γ, secreted by immune cells, decreases SLC7A11 expression thereby minimizing avenues for cystine uptake. Importantly, CTH may play a role in resisting ferroptosis in the presence of IFN-γ-secreting immune cells, owing to its role as an alternate source of cysteine for GBM cells.

#### HDAC2

Knockout of *HDAC2* was shown to synergize with chemotherapy in neuroblastoma [78]. In other studies, HDAC2 knockout in triple-negative breast cancer cells was shown to abrogate IFN-γ–dependent PD-L1 expression. The observed PD-L1 upregulation was shown to be reliant upon the JAK/STAT pathway upon IFN-γ binding the IFN-γ receptor. Furthermore, in HDAC knockout cells, p-JAK1, p-JAK2, and p-STAT were downregulated in comparison with HDAC-overexpressing cells [182, 183]. Overall, these findings may suggest a role for HDAC2 in conferring resistance to immune activity by driving exhaustion. Treatment with anti-PD-L1 may enhance NK cell effector function.

#### MET

Knockout of *MET* was shown, in a targeted CRISPR screen, to enhance treatment of neuroblastoma with chemotherapy [78]. Met inhibition has been shown to induce ATM phosphorylation, while ATM inhibition has been shown to potentiate type I IFN expression. Studies have also suggested that the increase in type I IFN production was driven by increased phosphorylation of TANK-binging kinase 1 (TBK1). In line with this, evidence supports the notion that type I IFN production can occur independently of the cGAS/STING signaling pathway. Pancreatic cancer cell growth was enhanced in ATM-deficient cells when mice maintained a functional immune system, although this effect was not observed in non-obese diabetic (NOD) scid γ (NSG) mice. This suggests that the type I IFN response may play a crucial role in supporting a robust immune response against cancer. Additional studies have revealed that ATM inhibition mediates heightened levels of PD-L1, while mice deficient in ATM demonstrated higher survival in response to anti-PD-L1 blockade [184]. Together, this evidence suggests a potential role of ATM in perpetuating immune exhaustion.

#### ZC3H71A

Limited evidence surrounding the role of *ZC3H7A*, shown to drive GBM resistance to TMZ through a targeted CRISPR screen in GBM, in driving tumor progression has been reported [64]. However, studies have so far suggested a unique immunomodulatory role of zinc finger CCCH-type containing (ZC3H)11 A in cancer. Among these, greater NK cell infiltration into tissues lacking ZC3H11A expression in comparison to ZC3H11A competent tumors, as well as significantly increased IFN-β secretion were both reported. Long noncoding ZC3H7A (LncZC3H7A), which has been suggested to be transcriptionally co-regulated with ZC3H7A expression, facilitated elevated levels of type I IFN in response to viral infections. MHC-I expression was also significantly increased in ZC3H11A-KO tumors [185, 186]. It is established that MHC-I generally directs inhibition of NK effector function. Nevertheless, findings have indicated that the presence of certain activating receptors, or combinations of coactivating receptors, on NK cell surface can potentiate NK effector function in MHC-I positive tumors [187–190]. Importantly, these findings suggest that *ZC3H11A* may enhance the effector function of NK cells, in part, in an IFN-β-dependent manner.

#### MLH1

*MLH1*, discovered from a targeted CRISPR screen in GBM, was shown to sensitize GBM to TMZ treatment [64]. In the context of EGFRvIII mutant GBM with aberrant MGMT response, MMR knockout promoted resistance to TMZ therapy [100]. Knockout of *MLH1* led to prolonged DNA damage following IR in 4T1 models. In addition, in a cGAS-dependent manner, MLH1 deficiency was found to trigger type I IFN expression by tumor cells [191]. As described, type I IFN mediate innate immunity, specifically NK circuits, which drive effector function, implicating *MLH1* in NK cell activation.

#### USP21

USP21 was identified as a dependency in GBM identified in an RNAi screen [101]. As discussed, FOXD1 expression was found to promote a MES GBM phenotype generally associated with greater glycolytic activity and lactate accumulation [101, 103]. Interestingly, tumors with low capacity for conversion of pyruvate to lactate had higher presence of NK cells [192]. These findings have suggested that MES GBM cells may be less susceptible to NK cell infiltration, via, in part, FOXD1 overexpression mediated by USP21.

#### CHMP2A

In a TCT screen in GBM cells, where GBM cells transduced with CRISPR KO libraries and co-cultured with NK cells, *CHMP2A* was found to sensitize GBM to NK cells. Extracellular vesicles (EVs) secreted by *CHMP2A* KO tumors were characterized by lower expression of MICA/B ligands [104]. Importantly, data showed significantly greater killing of human fibroblasts expressing MICA surface protein by NK cells in the presence of serum from soluble MICA^low^ patients as opposed to serum from soluble MICA^high^ patients. Moreover, the lack of cytotoxicity in MICA^high^ patients was determined to be in part due to an inherent inability for soluble MICA to activate NKG2D receptors [193]. Together, this suggests an immunosuppressive role of EVs secreted by *CHMP2A* WT cancer cells.

#### ARIH2 & CCNC

Both *ARIH2* and *CCNC* were identified in a TCT screen in NK cells. In this study, NK cells were engineered to KO *ARIH2* and *CCNC*, and co cultured with PATC148 cells. Data have suggested that this facilitated differential regulation of 21 enriched pathways compared to non-engineered controls. Some of the upregulated pathways in *ARIH2* and *CCNC* KO NK cells were cytokine receptor interaction, IFN response, and IL signaling pathways. These data suggest a role in *ARIH2* and *CCNC* in regulating the capacity for NK cells to respond to their environment in a tumor setting. Additionally, it was determined that STAT5, a member of the JAK/STAT pathway was enriched in *ARIH2* and *CCNC* KO NK cells further highlighting the importance of this pathway in both GBM and NK cells [107, 183].

## Therapeutic opportunities and challenges

Genetic screens carried out so far in GBM have enabled the identification of genes driving metabolism, growth and cell cycle, DNA-repair and immortality pathways. A critical aspect of genes identified through genetic screening efforts are their direct or indirect impact on cell surface receptor expression levels. Also, of importance are the interactions of these receptors with cancer directly or through the TME, which may also be impacted by oncogene expression. Furthermore, candidates identified through genetic screening studies may present specific vulnerabilities of cancer and its interaction with the immune system. These genetic vulnerabilities may shift competitive advantages in favor of NK cells. However, care should be taken when analyzing hits from genetic screens in cancer due to a strong contextual importance surrounding each finding. In the limited subspace of genetic screens specifically carried out in GBM; approaches have spanned a spectrum from comprehensive, genome-wide analyses to more focused investigations targeting druggable genetic candidates. In addition, incorporating factors such as chemotherapy or radiotherapy into genetic screening studies underscores the importance of contextualizing the genetic dependencies identified through these screens. For the most part, most of these screens have so far focused on the determination of perturbations that sensitize GBM or make it more resilient. Mutations in genes like *BRCA2* are well-established in promoting DNA damage and facilitating tumorigenesis in cancers such as breast cancer. However, *BRCA2* has also been shown to be essential for repairing DNA damage in GBM cells, contributing to their resistance to radiotherapy. [194] Importantly, the specific and often heterogeneous contexts surrounding genetic targets during the screening process may complicate clinical translation of preliminary findings.

A primary intent of this review was to highlight the genetic targets which have emerged in some of the various genetic screens in GBM. Then, subsequently, to highlight the intricate relationships between cancer and NK cells. Indeed, genetic vulnerabilities identified in cancer were found to have interconnected roles in modulation of NK and other immune cells. Among the studies discussed, relatively few were performed in NK cells in co-culture with GBM cells. The lack of other studies which probe genetic dependencies through CRISPR screening of NK cells in co-culture with GBM highlights challenges associated with this line of investigation. Among these is the difficulty in generating genetically-edited pNK cells, thereby limiting the quantity of cells available for genetic screens requiring gene library knockouts in NK cells [195]. Regardless, a deeper analysis of how the molecular implications of vulnerabilities or competitive advantages introduced into NK cells affect GBM may contribute to improved immunotherapies. This may, also, advance the general understanding of cancer progression and immune responses.

It is important to note that genes which may emerge as critical to GBM responses may also have a role on NK cells. One such gene, *LRP8*, is essential for NK cell function while also representing a genetic dependency in GBM [72, 137]. Non-specific targeting of *LRP8* could therefore be detrimental to healthy immune populations. To enhance tumor specificity and reduce off-target effects, targeted delivery systems may be employed. In this context, nanocapsules functionalized with angiopep-2—targeting LDL receptor-related protein 1 (LRP1)—have been developed to improve GBM-specific drug delivery. These nano-capsules achieved CRISPR KO efficiencies of 38% in-vitro and 64% in-vivo, although efficacy responses are yet to be determined [196, 197].

Another aspect driving future clinical translation of gene editing of immune cells is their amenability to genetic manipulation. Autologous NK cells sourced from patients, though safer and patient-specific, have been associated with poorer function and limited capacity for expansion when compared to allogenic NK cells. In response to clinical needs for large amounts of viable NK cells, alternative sources such as induced pluripotent stem cells, umbilical cord blood and cell lines (such as NK-92) have been evaluated both pre-clinically and clinically. [198] Pairing allogenic pNK and GBM cells, engineered to target hits emerging from CRISPR screens, has the potential to enhance therapeutic efficacy of NK cells.

To date, no clinical trials have directly evaluated therapeutic targets identified through CRISPR-based genetic screening in GBM; although several candidate genes such as *MAP4K4, E2F6*, and *UBE2N* have demonstrated preclinical promise, their translation into clinical settings remains at an early stage, highlighting the need for further in vivo validation and tumor-specific delivery strategies. Overall, targeting genes that sensitize GBM to NK cell-mediated cytotoxicity holds promise for enhancing immunotherapy efficacy. However, many of these hits also play critical roles in normal immune cell function, raising concerns about potential off-target effects and immune toxicity. To overcome these challenges, strategies such as tumor-specific delivery systems, combinatorial targeting approaches, and in vivo validation in immunocompetent models are essential to advance CRISPR-identified candidates toward clinical application.

## Further directions and clinical implementation

### Mechanistic elucidation

In this review we systematically analyzed and summarized multiple studies to connect findings from genetic screens in GBM with their role in modulation of NK cell function (Table 3). While these genes represent modulators of specific cellular processes in the context of GBM therapy, direct mechanistic connection between these genetic dependencies in GBM and NK functionality remain to be defined. As future studies advance toward functional validation of identified targets, it will be important to elucidate the underlying mechanisms driving these responses.

For instance, studies have shown higher NK presence in tumors to be associated with lower pyruvate to lactate conversion, an effect hypothesized to be mediated by overexpression of FOXD1 via USP21. However, the mechanistic link between USP21 knockout–mediated depletion of FOXD1 and the subsequent enhancement of NK cell infiltration into tumors remains to be elucidated. Similarly, in the context of *CSE*, there is currently no direct evidence supporting its role in modulating resistance to ferroptosis in response to IFN-γ secretion by NK cells. Although such a mechanism has been demonstrated in the context of CD8⁺ T cells affecting GBM cell viability, direct investigations involving *CSE*-deficient GBM cells and NK cell interactions could provide mechanistic validation for the findings observed in CRISPR-based screening studies.

### Technological development

Recent technological advancements have significantly enhanced genetic screening in NK cells. A major challenge has, for a long time, been the difficult nature of NK cell engineering. Studies have reported that due to anti-viral mechanisms characteristic of NK cell innate immune responses, viral transduction efficiency is relatively low. These findings have prompted identification of viral envelopes with greater tropism for NK cells such as the baboon envelope pseudo-typed lentiviral vector (BaEV-LV) [199]. Genome-wide libraries, such as the Brunello library with its 77,441 sgRNAs [200], require efficient delivery systems to ensure adequate representation across target cells. Enhancing viral affinity for the target cell is therefore not only desirable but essential, as it reduces the number of cells needed to accurately represent the library [201]. This becomes even more critical in high-dimensional, combinatorial screening approaches. Technological advances—such as the selection and modification of viral envelopes—further support these complex strategies by improving delivery efficiency and expanding the scope for therapeutic exploration. Innovations such as CRISPR interference (CRISPRi) and activation (CRISPRa) have further allowed gene modulation without permanent genomic disruption, improving safety and specificity. Finally, innovations in electroporation technology—such as next-generation pulse generators—have significantly increased transfection efficiency and cell viability in NK cells. Mechanized platforms for automated liquid handling, high-throughput screening, and real-time cell analysis now allow for more scalable and reproducible screens.

### Clinical trials

**Fig. 3:**
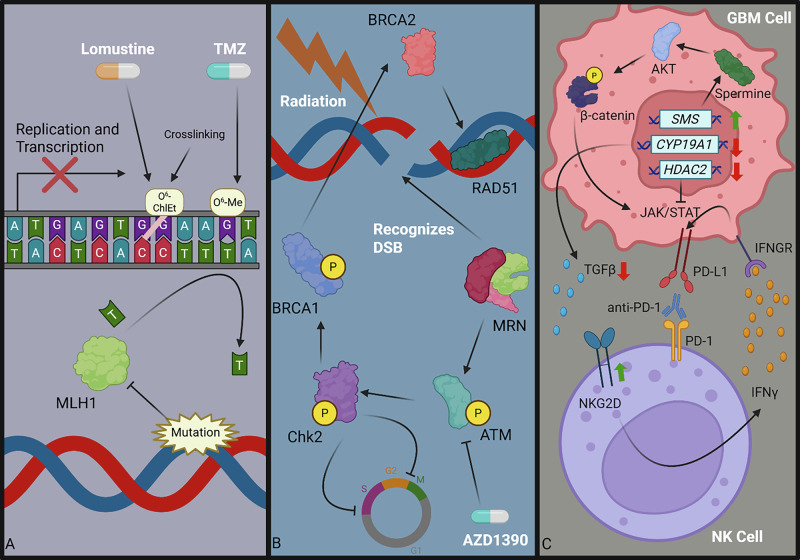
Interplay between genetic driver genes identified in genetic screens in GBM and therapeutic strategies in clinical trials. **A** Mechanism of action of Lomustine and TMZ combination therapy currently being evaluated in clinical trials (NCT05095376). Lomustine induces the formation of O6-ChlEtG adducts on guanine residues, which can facilitate interstrand crosslinking with cytosine, thereby obstructing DNA replication and transcription [203–205]. In contrast, TMZ primarily induces O6-MeG lesions, which, in the context of MMR proficiency, can trigger futile repair cycling. However, in many TMZ-resistant tumors, this effect is attenuated due to the high prevalence of MLH1 mutations, resulting in MMR deficiency [99, 100]. **B** Mechanism of action of ATM inhibition with AZD1390 currently being evaluated in clinical trials (NCT06894979). AZD1390 targets the DNA damage response activated by radiotherapy-induced DSBs. The MRN complex recognizes DSBs and recruits ATM, which phosphorylates Chk2 to initiate cell cycle arrest and DNA repair [58, 59, 206]. Chk2 further facilitates BRCA1 and BRCA2 recruitment, promoting RAD51 localization to damage sites and enabling HR sensitivity to radiotherapy [207, 208]. **C** Role of anti-PD1 antibody in enhancing NK cell function against GBM currently being evaluated in clinical trials with pembrolizumab (NCT06556563). Elevation of *SMS* drives AKT dependent, β-catenin phosphorylation which drives PD-L1 upregulation via the JAK/STAT pathway in GBM [147]. *CYP19A1* depletion drives decreased TGF-β levels which are correlated with upregulation of NKG2D, enhancing cytotoxic capacity of NK cells [122, 124]. Cytotoxicity of NK cells, specifically IFN-γ production, elevates JAK/STAT-dependent expression of PD-L1 regulated by *HDAC2* in GBM [179, 180].

Many of the genes which have been discussed in this review have importance and underlying implications in ongoing clinical trials in GBM. And though no ongoing trials target specific genes identified in screening studies, several clinical investigations implicate genetic dependencies identified in genetic screens. An ongoing phase III trial in MGMT-methylated GBM is evaluating efficacy of adding lomustine to standard therapy with TMZ and radiation (NCT05095376). This trial is based on prior evidence indicating that, in MGMT-deficient tumors, functional MMR can lead to futile repair cycling in response to O6-methylguanine lesions induced by TMZ, ultimately resulting in GBM cell death [99, 100]. Moreover, prevalence of methylated MGMT and mutations in genes encoding MMR machinery were observed in recurrent tumors previously treated with TMZ [202]. Unlike TMZ, the alkylating agent lomustine generates O6-chloroethylguanine (O6-ChlEtG) adducts. Through subsequent molecular rearrangement, this lesion can form interstrand crosslinks with cysteine residues. When such crosslinks occur between complementary DNA strands, they can impede both DNA replication and transcription [203–205]. Addition of lomustine with TMZ and radiation thereby presents an additional layer of DNA damage potentially detrimental to rapidly dividing GBM cells (Fig. 3).

A different phase I trial is underway evaluating administration of ATM inhibitor AZD1390 in combination with radiotherapy in glioma patients (NCT06894979). As we have discussed, MRN has been associated with the capacity to effectively mediate DSB repair. It has been well established that in response to DNA damage, MRN facilitates upregulation of p-ATM. Studies have also suggested that in the presence of DNA damage and competent MRN, p-ATM and p-Chk2 levels were elevated [58, 206]. Separately, phosphorylation of breast cancer type 1 susceptibility protein (BRCA1) was shown to be mediated by active Chk2 for competent HR of DSBs measured by RAD51 foci [207], while RAD51 recruitment was shown to depend on functional BRCA2 [208]. Therefore, this clinical study implicates both the MRN component Mre11a and BRCA2, further underlying the importance of analyzing CRISPR screens to strategically pursue rational therapies in GBM (Fig. 3).

Additional efforts have been made to pursue concurrent treatment with TMZ and pembrolizumab, an anti PD-1 antibody, as a strategy to delay immune exhaustion (NCT06556563) [148]. As was the case of *SMS*, discovered in a targeted CRISPR screen, spermine increased PD-L1 via AKT-dependent phosphorylation of β-catenin [79, 147]. The gene *CYP19A1*, identified in an OCT perturb-seq screen, was associated with increased tumor PD-L1 levels, potentially through concomitant decrease of TGF- β in the TME and upregulation of activating receptor NKG2D on NK cells during CYP19A1 inhibiton [40, 122, 124]. Finally, depletion of HDAC2, corresponding to *HDAC2*, identified in a targeted CRISPR screen, led to decreased IFN-γ-dependent PD-L1 expression with lower phosphorylation of proteins JAK1/2 and STAT^78182183^. In this context, this clinical trial reflects multiple genetic dependencies identified in CRISPR screens in GBM, further emphasizing the potential of validating screening outcomes toward actionable therapeutic targets (Fig. 3).

### Biomarker validation

An additional challenge with the translation of therapeutic targets identified in genetic screens resides, in some capacity, in the heterogeneity present even within the genomic landscape of a specific tumor type.

Typically, top biomarker candidates identified from screening studies will be validated through a combination of secondary focused screens and individual gene perturbation studies which include independent sgRNAs, rescue experiments, and functional assays—such as proliferation, apoptosis, or immune cell co-culture—to confirm their role in tumor biology. Further validation may involve in vivo models and correlation with patient data to assess clinical relevance and predictive potential. Specifically, to aid in translating the findings from CRISPR screens into clinically relevant therapies, patient scRNA-seq data sets can be used to correlate hits with clinical features such as OS and progression free survival (PFS).

### Policy

Genomic editing introduces questions which highlight considerations of regulatory frameworks, access and scope of such studies. These findings carry significant policy implications, underscoring the need for updated regulatory frameworks that address ethical considerations, data privacy, and access to emerging gene-editing technologies. Specifically, the use of genetic editing in human cells raises complex ethical considerations, particularly regarding off-target effects, potential germline modifications, and the long-term safety of edited cells in therapeutic contexts. Second, as genetic screens generate vast amounts of sensitive genomic data, policies must ensure robust data privacy protections and prevent misuse or unauthorized access.

The recommendation that novel therapeutics have both grounding in patient datasets and thorough validation prior to being pursued in human patients may be one of the first steps toward the efficient clinical application of these targets [209, 210].

## Conclusions

Genetic signatures underlying GBM development, progression and response to therapy hold important implications for understanding GBM pathology. In that context, targeted and unbiased genetic screens are powerful tools for establishing the functional significance of specific genetic alterations. This may aid identifying novel therapeutic targets and elucidating the molecular mechanisms driving GBM progression and resistance to conventional treatments. The genetic screens in GBM have identified mutations commonly found across various cancers, such as *TERT* dependency. Additionally, genetic dependencies have been discussed that synergize with mutations unique to GBM, such as *MLH1* driving TMZ sensitivity, partially due to the epigenetic reprogramming characteristic of GBM. More generally, this review not only presents genetic dependencies typically present in most tumors but also reveals unique characteristics of GBM in comparison to other tumors.
